# Supplementary material for: The interactome of intact mitochondria by cross-linking mass spectrometry provides evidence for coexisting respiratory supercomplexes
Source: Mol Cell Proteomics. 2017 Dec 8;17(2):216–32. doi: 10.1074/mcp.RA117.000470 (PMC5795388; doi:10.1074/mcp.RA117.000470)
Supplement: Supplemental Data [file supp_RA117.000470_133922_0_supp_23978_fzffwf.zip › spectra_annotation/mito_DR_spectra_annotation/201-1-1-1-9-1.pdf]

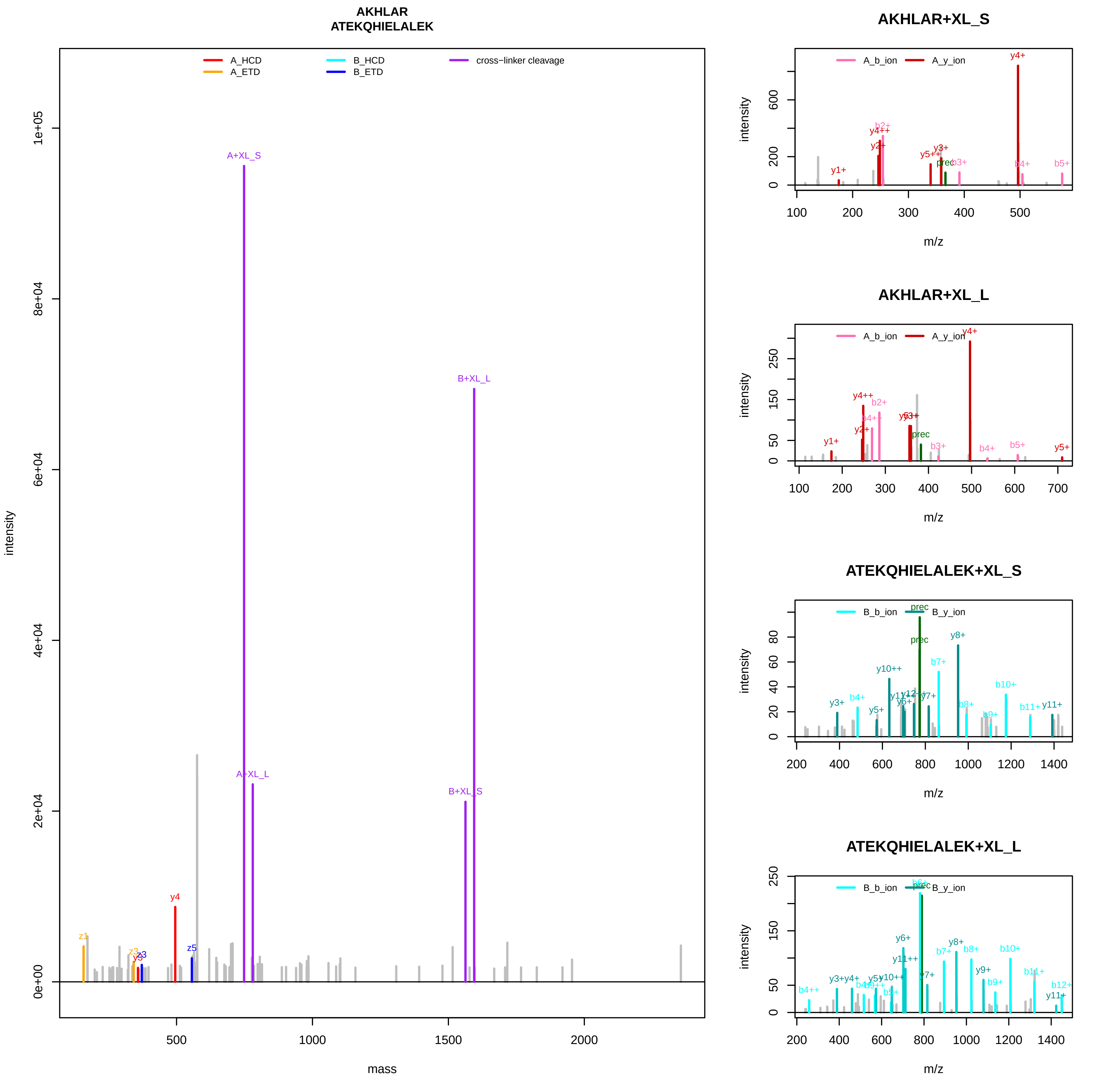
AKHLAR
ATEKQHIELALEK
A_HCD
A_ETD
B_HCD
B_ETD
cross−linker cleavage
A+XL_S
B+XL_L
A+XL_L
B+XL_S
y4
z1
z3
y3
z3
z5
intensity
mass
0e+00
2e+04
4e+04
6e+04
8e+04
1e+05
500
1000
1500
2000
AKHLAR+XL_S
A_b_ion
A_y_ion
y4+
b2+
y4++
y2+
y1+
y5++
y3+
prec
b3+
b4+
b5+
m/z
AKHLAR+XL_L
y4++
b2+
b4++
y2+
y1+
y5+
b3+
b4+
b5+
ATEKQHIELALEK+XL_S
B_b_ion
B_y_ion
y3+
b4+
y5+
y10++
y6+
y8+
b7+
y7+
b8+
b9+
b10+
b11+
y11+
ATEKQHIELALEK+XL_L
b4++
y3+
y4+
y5+
y10++
y6+
y11++
b7+
y8+
b8+
y9+
b9+
b10+
b11+
b12+
y11+
